# Adverse dermatoneurological events and impacts on daily activities of patients with gastrointestinal neoplasms undergoing chemotherapy

**DOI:** 10.1590/0034-7167-2022-0161

**Published:** 2023-01-30

**Authors:** Rafaela Moreira da Silva Canille, Maria Helena Pinto, Katia Jaira Galisteu, Rildo César Czorny, Luana Gaino Bertolazzi, Tamara Veiga Faria

**Affiliations:** I Hospital de Base de São José do Rio Preto. São José do Rio Preto, São Paulo, Brazil; II Faculdade de Medicina de São José do Rio Preto. São José do Rio Preto, São Paulo, Brazil; III União Faculdade dos Grandes Lagos. São José do Rio Preto, São Paulo, Brazil; IV Serviço Nacional de Aprendizagem Comercial. Barretos, São Paulo, Brazil; V Faculdade Ceres. São José do Rio Preto, São Paulo, Brazil

**Keywords:** Adverse Events, Nursing Oncology, Antineoplastic, Gastrointestinal Neoplasms, Activities of Daily Living, Eventos Adversos, Enfermería Oncológica, Antineoplásicos, Neoplasias Gastrointestinales, Actividades Cotidianas, Eventos Adversos, Enfermagem Oncológica, Antineoplásicos, Neoplasias Gastrointestinais, Atividades Cotidianas

## Abstract

**Objective:**

to associate the presence and grading of adverse dermatoneurological events (peripheral neuropathy and hand-foot syndrome) and the interference in the activities of daily living of patients with gastrointestinal neoplasms undergoing systemic antineoplastic treatment.

**Method:**

this is a longitudinal, prospective study, using instruments to assess hand-foot syndrome and peripheral neuropathy.

**Results:**

there were 36 patients: 66.7% diagnosed with colon cancer and 83.2% on combination therapy. From cycle 5 onwards, all of them had hand-foot syndrome, with a majority of grade 1, unrelated to interference in activities of daily living. Regarding peripheral neuropathy, there was a moderate to strong correlation from cycle 1 of treatment.

**Conclusion:**

peripheral neuropathy negatively affects activities of daily living. The monitoring of dermatoneurological events by oncology nurses contributes to the clinical practice of nursing and subsidizes the development of advanced practice in the country.

## RESUMO

**Objetivo:**

associar a presença e a graduação de eventos dermatoneurológicos adversos (neuropatia periférica e síndrome mão-pé) e as interferências nas atividades da vida diária de pacientes com neoplasias gastrointestinais em tratamento antineoplásico sistêmico.

**Método:**

trata-se de um estudo longitudinal, prospectivo, com a utilização de instrumentos de avaliação da síndrome mão-pé e da neuropatia periférica.

**Resultados:**

foram 36 pacientes: 66,7% com diagnóstico de câncer de cólon e 83,2% em terapia combinada. A partir do ciclo 5, todos apresentaram a síndrome mão-pé, com maioria de grau 1, não relacionada com a interferência nas atividades da vida diária. Em relação à neuropatia periférica, houve a correlação de intensidade moderada a forte desde o ciclo 1 de tratamento.

**Conclusão:**

a neuropatia periférica afeta negativamente as atividades da vida diária. O acompanhamento dos eventos dermatoneurológicos pelo enfermeiro oncológico contribui para a prática clínica da enfermagem e subsidia o desenvolvimento da prática avançada no país.

## RESUMEN

**Objetivo:**

asociar la presencia y gradación de eventos dermatoneurológicos adversos (neuropatía periférica y síndrome mano-pie) y la interferencia en las actividades de la vida diaria de pacientes con neoplasias gastrointestinales en tratamiento antineoplásico sistémico.

**Método:**

se trata de un estudio longitudinal, prospectivo, utilizando instrumentos para evaluar el síndrome mano-pie y la neuropatía periférica.

**Resultados:**

hubo 36 pacientes: 66,7% con diagnóstico de cáncer de colon y el 83,2% en terapia combinada. A partir del ciclo 5, todos presentaban síndrome mano-pie, en su mayoría grado 1, no relacionado con interferencia en actividades de la vida diaria. Con respecto a la neuropatía periférica, hubo una correlación de moderada a fuerte desde el ciclo 1 de tratamiento.

**Conclusión:**

la neuropatía periférica afecta negativamente las actividades de la vida diaria. El seguimiento de los eventos dermatoneurológicos por parte de los enfermeros de oncología contribuye a la práctica clínica de enfermería y subsidia el desarrollo de la práctica avanzada en el país.

## INTRODUCTION

Gastrointestinal tract (GIT) neoplasms represent the second most frequent oncological diseases in both sexes^(1)^. Patients diagnosed with these neoplasms have biopsychosocial changes caused by the illness and by the location of the disease, which can be potentiated due to interference in the processes of food intake, digestion and absorption^(2)^.

In this context, one of the treatment modalities of gastrointestinal neoplasms is systemic antineoplastic chemotherapy, in which chemical substances are used, alone or in combination^(3)^. These substances act in different phases of the cell cycle, and can reach normal cells, causing several adverse events, such as dermatoneurological changes (peripheral neuropathy (PN) and hand-foot syndrome (HFS))^(3)^, mainly in antineoplastic stemunatoric and fluopyrimidine, widely used in the therapeutic protocols of these neoplasms^(4)^.

Such adverse events are present in most patients undergoing cancer treatment and are present in the routine of cancer nurses. Thus, the oncology specialty is a major challenge for nurses, as, in addition to knowing the pathophysiology of the oncological disease, they must develop skills to perform patient care that minimizes their symptoms and preserves their quality of life.

A major challenge for professionals is chemotherapy-induced peripheral neuropathy (CIPN), because, despite being quite frequent as an adverse event related to antineoplastic treatment in a cytotoxic agent, its pathogenesis is not completely defined. Studies infer that antineoplastic chemotherapy drugs are able to gradually damage sensory neurons and subsequently irreversibly degenerate their structure^(5)^. The symptomatology of PN is related to the type of nerve fiber affected, resulting in sensory and motor signs and symptoms. Sensory alterations, such as paresthesia and dysesthesia, are the most frequent due to damage to sensory fibers, while damage to motor fibers promotes changes in muscle strength^(6)^.

When compared to PN, which is also part of the routine of an anticancer drug infusion center, PMS is also known as palmoplantar erythrodysesthesia, acral erythema or Burgdorf reaction. It is a toxic skin reaction related to the high sensitivity of skin tissues to the action of antineoplastic drugs^(7)^, which can mediate a toxic effect on basal keratinocytes, leading to basal vacuolar degeneration and complete necrosis of the epidermal layer^(8)^. It is characterized by palmoplantar numbness, tingling or burning pain associated with erythema with or without swelling and cracking or scaling. Other histological findings found were inflammation at the dermoepidermal junction and dilation of blood vessels^(9)^.

Adverse dermatoneurological events such as PN and HFS are most often accompanied by pain and may limit activities of daily living (ADL)^(10)^, such as walking, picking up objects, driving, working, sleeping, participating in leisure activities, exercising, relating to people, writing, household activities, activities involving manual dexterity and sexual activity^(11)^.

Moreover, considering the possible interference of such adverse events in ADL, patient treatment becomes the focus of medical care and information and continuous interaction of the nursing team, including the coexistence of patients, from diagnosis, with the possibility of recurrence or progression of illness^(12-13)^. Therefore, nursing interventions aim to improve well-being, and may be aimed at controlling pain, fatigue and other adverse events related to anticancer therapy. And the effectiveness of these actions can contribute, in addition to the relief of signs and symptoms, to improve the therapeutic response and safety in drug administration^(12-13)^.

Nursing interventions, including decision-making for better treatment option, are even more valued when considering the continuous increase in new oncological therapeutic options, making it necessary to continuously involve these professionals with knowledge about the management of signs and symptoms and consequent improvement in patients’ quality of life ^(13-14)^.

Considering that the adverse events of systemic antineoplastic treatment can cause damage to ADL, and that, in their daily practice, the oncology nurse often finds it difficult to measure such events as HFS and CIPN, this study aims to associate the presence and grading of adverse dermatoneurological events (peripheral neuropathy and hand-foot syndrome) and interference in the ADL of patients with gastrointestinal neoplasms undergoing systemic antineoplastic treatment.

## OBJECTIVE

To associate the presence and grading of adverse dermatoneurological events (peripheral neuropathy and hand-foot syndrome) and interference in ADL of patients with gastrointestinal neoplasms undergoing systemic antineoplastic treatment.

## METHOD

### Ethical aspects

This study was approved by the Research Ethics Committee of School of *Faculdade de Medicina de São José do Rio Preto*/SP. All study procedures complied with the precepts of Resolution 466/12 of the Brazilian National Health Council.

### Study design and site

This is an observational, longitudinal and prospective study, guided by the STROBE tool, which took place in the Chemotherapy Unit (CU) of a university hospital, with a quaternary scope, in the countryside of the state of São Paulo, Brazil.

### Inclusion and exclusion criteria

The possible candidates for the study were selected through the medication dispensing report, issued by the hospital system. Patients with gastrointestinal neoplasms, of both sexes, aged at least 18 years, beginners in both monotherapy and combined therapies, inserted in the CAP (Capecitabine), CAPOX (Capecitabine and Oxaliplatin), EOX (Epirrubicin, Oxaliplatin and Capecitabine), CAP + BEV (Capecitabine and Bevacizumab) and CAP + GENC (Capecitabine and Gemcitabine), indicated by oncologists and administered in the oncology service routine, were included in this study. The cases in which the dispensed capecitabine monotherapy was not delivered directly to patients, in which there were deaths, therapeutic change and discontinuation of treatment in the first cycle were excluded.

Data were collected by the same researcher to reduce the risk of data collection bias through interview at the time of the nursing consultation (screening consultation before drug infusion), for a period of eight months, from July 2016 to March 2017. The sample consisted of patients who could have at least two monitored and/or followed-up cycles, and the number of cycles was defined by the therapeutic purpose and by the antineoplastic treatment protocol. Participants answered the sociodemographic form and the HFS and CIPN questionnaires before the start of treatment and before the release of subsequent cycles, with the interval of treatment cycles performed every 21 days.

And, for this data collection, the following were used: (i) form of sociodemographic characteristics (gender, age, marital status, religion, work situation, comorbidities, use of alcoholic beverages and use of tobacco/tobacco, in addition to the date of the biopsy for diagnostic confirmation, location of neoplasms, therapeutic purpose and protocol of anticancer treatment); (ii) visual analysis instrument to classify HFS, based on the signs and symptoms presented; and (iii) CIPN assessment/aggravation tool that does not distinguish sensory from motor peripheral neuropathy^(15)^. In the adapted version of the tool, nine specific neuropathic symptoms were evaluated (numbness and tingling in the hands, numbness and tingling in the feet, sensitivity to cold, neuralgia, muscle/joint pain, weakness in the arms/hands/legs/feet, and balance problems) and how much these symptoms interfere with the 13 ADL items (dressing, walking, picking up and holding objects, driving, working, sleeping, participating in leisure activities, practicing physical exercises, relating to people, writing, performing activities households and enjoy life), through a numerical scale, with high scores (the higher the score, the higher the frequency and greater interference in ADL).

The HFS grading was based on the Common Terminology Criteria for Adverse Events (CTCAE), version 5.0, which is classified as grade 1, characterized by minimal skin changes or non-painful dermatitis (erythema, edema or hyperkeratosis); grade 2, skin changes with pain (blisters, light scaling, sores, edema or hyperkeratosis), limiting daily activities; and grade 3, severe skin changes such as severe scaling, constant pain, and severe limitation of self-care^(16)^.

Study participants, as established by clinical practice, received educational guidelines on the treatment performed, namely: i - use of comfortable shoes; ii - attention and care when handling sharp objects; iii - wearing gloves when handling cleaning products or chemicals; iv - do not open the refrigerator without protective gloves; v - avoid temperature extremes and keep hands and feet warm; vi - moisturize hands and feet at least three times a day (medical staff usually recommend urea-based moisturizing cream); vii - use handrails or walkers when walking, rooms and passages of the house should be well lit and free of rugs and rest in a comfortable place in case of more intense pain.

### Analysis of results, and statistics

The data was received and registered in Microsoft Excel. Subsequently, they were imported into IBM-SPSS Statistics, version 27 (IBM Corporation, NY, USA), for exploratory data analysis and comparative analysis between groups. Exploratory data analysis included descriptive statistics, means, medians, standard deviation, quartiles, minimum and maximum value for numerical variables and, finally, number and proportion for categorical variables. Descriptive statistics, histogram and boxplot graphs and the specific test for the theoretical assumption of normality Kolmogorov-Smirnov were considered for the analysis of the behavior of continuous variables. Comparative analysis of ADL between groups with and without HFS was performed using the Mann-Whitney test for cycles with a sufficient number of cases.

Correlation analysis between ADL and CIPN was performed using Pearson’s correlation coefficient for all treatment cycles. Pearson’s correlation coefficient values were accepted using the scale of values +1 to -1. When the value is close to +1, perfect positive linear correlation is assumed; when the value is close to -1, a perfect negative linear correlation is assumed; values close to zero indicate the absence of correlation. More than the p-value, it is suggested to take into account the value of the coefficient. The following values can be assumed: |≤0.25| = absence of correlation; |0.26 - 0.50| = weak correlation; |0.51 - 0.75| = moderate correlation; and |>0.75| = strong correlation^(17)^. All tests were two-tailed, and values of p < 0.05 were considered statistically significant.

## RESULTS

We selected 144 possible candidates for the study and of these, 108 were excluded (100 patients who were not newcomers, two due to death, two due to therapeutic change, two due to discontinuation of treatment and two in which the medication was dispensed to the family), and the final sample comprised 36 patients. Participants’ age ranged from 30 to 85 years, with a mean of 59 ± 12.7 years. There was a predominance of men (58.3%). Most had elementary school (52.8%). Regarding marital status, 75% were married and Catholicism was the prevalent religion (61.1%). Participants’ work situation was classified as retired (41.7%), domestic workers (22.2%), those on sick leave (19.4%), self-employed (13.9%) and employees (2.8%). The majority had no comorbidities (52.8%) and 88.9% said they did not consume alcohol.

It was identified that the predominant gastrointestinal neoplasms were colon (66.7%) and most classified as adjuvant (47.2%) or palliative (47.2%) treatment. Combined therapy with CAPOX (Capecitabine + Oxaliplatin) (83.2%) represented the most used therapeutic protocol, as shown in Table 1.

**Table 1:** Location of gastrointestinal tract neoplasms, purpose of treatment and antineoplastic protocol, São José do Rio Preto, São Paulo, Brazil, 2016

	n (%)
Location	
Colon	24 (66.7)
Stomach	7 (19.4)
Rectum	3 (8.3)
Esophagus	2 (5.6)
Treatment purpose	
Adjuvant	17 (47.2)
Palliative	17 (47.2)
Neoadjuvant	2 (5.6)
Antineoplastic protocol	
Capecitabine + Oxaliplatin	30 (83.2)
Capecitabine + Bevacizumab	2 (5.6)
Capecitabine	2 (5.6)
Capecitabine + Gemcitabine	1 (2.8)
Capecitabine + Epirubicin + Oxaliplatin	1 (2.8)

It was observed that from cycle 5, all patients presented HFS, with predominance of grade 1 (Table 2).

**Table 2:** Proportion of patients with the presence of hand-foot syndrome and their grading during the eight treatment cycles among patients analyzed in each cycle, São José do Rio Preto, São Paulo, Brazil, 2016

		SMP				
**Cycles**	**n**	**Absent**	**Present**	**Grade 1**	**Grade 2**	**Grade 3**
Cycle 1	36	35 (97.2)	1 (2.8)	1 (2.8)	0 (0.0)	0 (0.0)
Cycle 2	31	22 (71.0)	9 (29.0)	8 (25.8)	1 (3.2)	0 (0.0)
Cycle 3	30	8 (26.7)	22 (73.3)	22 (73.3)	0 (0.0)	0 (0.0)
Cycle 4	30	3 (10.0)	27 (90.0)	25 (86.2)	2 (2.7)	0 (0.0)
Cycle 5	26	0 (0.0)	26(100.0)	24 (92.3)	1 (3.8)	1 (3.8)
Cycle 6	22	0 (0.0)	22 (100.0)	19 (86.4)	2 (9.1)	1 (4.5)
Cycle 7	13	0 (0.0)	13 (100.0)	12 (92.3)	0 (0.0)	1 (7.7)
Cycle 8	5	0 (0.0)	5 (100.0)	4 (80.0)	0 (0.0)	1 (20.0)

*HFS: hand-foot syndrome.*

The comparative analysis between ADL and the presence of HFS showed that among the analyzed cycles (cycles two, three and four), there was no significant relationship between the presence of HFS and interference in ADL in each treatment cycle according to Table 3. It is worth mentioning that it was not possible to obtain the inferential statistics in cycles 1, 5, 6, 7 and 8, due to the number of patients who were starting the respective treatment cycle with the presence of HFS; in other words, for comparison in cycle 1, only one patient with HFS, and for comparison in cycles 5, 6, 7 and 8, all patients with HFS. Therefore, there was no variability between the presence of HFS and ADL score.

**Table 3:** Comparative analysis of activities of daily living, according to the presence of hand-foot syndrome, São José do Rio Preto, São Paulo, Brazil, 2016

Cycle	Absent HFS				Present HFS			*p* value
	ADL score				ADL score			
	n	Median Min; Max	SD		n	Median Min; Max	SD	
Two	22	13.0	14.2		9	21.0	27.9	0.176
		0.0 ; 62.0				11.0 ; 95.0		
Three	8	21.0	12.29		21	24.0	11.64	0.864
		0.0 ; 38.0				10.0 ; 58.0		
Four	3	19.0	7.02		27	22.0	15.41	0.406
						0.0 ; 78.0		

*ADL: activity of daily living; HFS: hand-foot syndrome; SD: standard deviation; Min - minimum; Max - max.*

**Figure 1:**
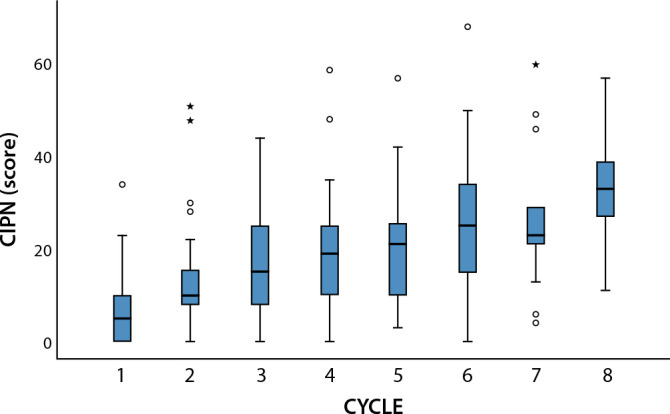
Proportion of patients with chemotherapy-induced peripheral neuropathy during the eight treatment cycles, São José do Rio Preto, São Paulo, Brazil, 2016

Next, Figure 1 shows the proportion of CIPN during treatment cycles. A gradual increase in the intensity of neuropathic symptoms was observed during the cycles performed by patients.

Below, in Table 4, according to Pearson’s analysis, it was identified that neuropathic symptoms correlate with moderate (0.51 - 0.75) to strong (>0.75) ADL in the first cycle of treatment, and in cycles 7 and 8, the highest occurrence of strong intensity correlation is observed, thus suggesting the cumulative toxicity of the proposed treatment.

**Table 4:** Pearson correlation analysis between activity of daily living and chemotherapy-induced peripheral neuropathy in each cycle, São José do Rio Preto, São Paulo, Brazil, 2016

CIPN		Activities of daily living in cycles							
		Cycle 1 ADL	Cycle 2 ADL	Cycle 3 ADL	Cycle 4 ADL	Cycle 5 ADL	Cycle 6 ADL	Cycle 7 ADL	Cycle 8 ADL
Cycle 1	Coefficient *r*	0.801^**^	0.633^*^	0.571^*^	0.602^*^	0.636^*^	0.541^*^	0.674^*^	0.846^**^
	*p* value	**< 0.001**	**< 0.001**	**0.001**	**< 0.001**	**0.001**	**0.011**	**0.023**	0.071
	n	35	30	29	30	24	21	11	5
Cycle 2	Coefficient *r*	0.576^*^	0.69^*^	0.618^*^	0.743^*^	0.662^*^	0.749^*^	0.93^**^	0.985^**^
	*p* value	**0.001**	**< 0.001**	**0.001**	**< 0.001**	**0.001**	**0.001**	**0.001**	0.112
	n	31	31	26	25	20	16	8	3
Cycle 3	Coefficient *r*	0.538^*^	0.53^*^	0.692^*^	0.719^*^	0.667^*^	0.761^**^	0.746^*^	0.971^**^
	*p* value	**0.002**	**0.005**	**< 0.001**	**< 0.001**	**0.001**	**< 0.001**	**0.034**	**0.029**
	n	30	26	29	26	21	19	8	4
Cycle 4	Coefficient *r*	0.412	0.534^*^	0.785^**^	0.721^*^	0.640^*^	0.588^*^	0.675^*^	0.911^**^
	*p* value	**0.024**	**0.006**	**< 0.001**	**< 0.001**	**0.001**	**0.006**	**0.023**	**0.031**
	n	30	25	25	30	23	20	11	5
Cycle 5	Coefficient *r*	0.629^*^	0.697^*^	0.737^*^	0.686^*^	0.744^*^	0.705^*^	0.770^**^	0.879^**^
	*p* value	**0.001**	**0.001**	**< 0.001**	**< 0.001**	**< 0.001**	**0.002**	**0.015**	0.121
	n	23	18	19	22	22	17	9	4
Cycle 6	Coefficient *r*	0.585^*^	0.721^*^	0.714^*^	0.670^*^	0.583^*^	0.609^*^	0.731^*^	0.804^**^
	*p* value	**0.004**	**0.001**	**0.001**	**0.001**	**0.009**	**0.003**	**0.016**	0.101
	n	22	17	19	20	19	21	10	5
Cycle 7	Coefficient *r*	0.677^*^	0.651^*^	0.770^**^	0.663^*^	0.632^*^	0.760^**^	0.763^**^	0.914^**^
	*p* value	**0.011**	**0.042**	**0.015**	**0.014**	**0.037**	**0.004**	**0.006**	**0.030**
	n	13	10	9	13	11	12	11	5
Cycle 8	Coefficient *r*	0.738^*^	0.890^**^	0.968^**^	0.752^**^	0.879^**^	0.825^**^	0.745	0.909^**^
	*p* value	0.155	0.301	**0.032**	0.143	0.121	0.085	0.255	**0.032**
	n	5	3	4	5	4	5	4	5

ADL: activity of daily living; CIPN: chemotherapy-induced peripheral neuropathy; (^*^) moderate correlation; (^**^) strong correlation.

## DISCUSSION

In literature, studies propose to assess the interference in ADL using questionnaires on quality of life (QoL). In the present study, we chose to associate the grading of adverse events and the interference in ADL through specific tools for the analysis of these events, in addition to relating them to the interferences in the daily habits of cancer patients and studying how this information contribute to qualify the nursing team’ work in oncology.

In the present study, it is observed that both in adjuvant and palliative treatment, most intestinal neoplasms are treated with platinum analogues, specifically oxaliplatin, according to information from an international study^(18)^. The probable dermatoneurological events related to this therapy and the use of a tool for nursing interventions are highlighted, valuing their actions related to the maintenance and improvement of cancer patients’ QoL. This study shows that dermatoneurological adverse events intensify when the combination of two neurotoxic drugs occurs, as in the drug protocol, CAPOX, corroborating the findings of other scientific analyzes ^(19)^, and adding the importance of the theme to cancer nurses.

Vulnerability to chemotherapy is different between the lines of antineoplastic chemotherapy. When it comes to palliative therapy, the risk of drug-related toxicity becomes more frequent^(20)^, which contradicts the findings of this study, as a similarity was identified between toxicity in the purpose of adjuvant and palliative treatment. This result helps to strengthen nurses’ concept that care and attention to cancer patients undergoing treatment should be intensified from the beginning of the process and regardless of the presence of metastatic disease.

As for HFS grading and interference in ADL, a survey carried out with Dutch oncologists shows that the majority of respondents report that grade 2 (85%) and grade 3 (97%) are the ones that have relevant impacts on patients’ QoL^(8)^. On the other hand, in the current study, we evidenced nursing care activities as an adjunct to cancer treatment, and HFS grade 1 was the most frequent and not directly related to interference in patients’ daily habits.

On the other hand, regarding treatment time and PN, a multicenter study in patients with colorectal cancer, treated with combined therapy, points out that grade 2 was more common in patients treated for 6 months than in patients treated for 3 months^(18)^. This research corroborates this information and also adds that, according to the PN intensity classification, this event may manifest early moderately to strongly interfering in ADL of patients who, in addition to this toxicity, may also present symptoms related to neoplastic involvement of the primary site of the oncological disease. Therefore, oncology nurses must identify the presence of PN, intensify educational activities for these patients and monitor the intensity of this event, thus contributing to the maintenance of the life habits of its clients.

A probable explanation for these identified data is that the intensity of PN is proportional to the cumulative dose of antineoplastic and sensory symptoms, accompanied by pain, can be observed for a long time after the end of treatment^(4)^. Also, according to the tool used, negative influences on ADL are defined by difficulties in walking, altered balance, impairment in fine motor movements (typing, driving, writing, working), difficulty feeling tiny objects and buttoning clothes^(21-22)^.

The result of our study that should be highlighted refers to nursing interventions that aim to identify possible adverse events and consequently propose actions: i - use of specific tools to assess adverse events related to antineoplastic therapy; ii - nursing interventions that prevent dose adjustment or interruption of anticancer treatment and maintenance or improvement of lifestyle. In a sense, the proposal is that the nursing team assist in increasing the overall survival of cancer patients. Therefore, this is a result that values nursing actions, as they may have the purpose of proposing prophylactic measures for two very frequent adverse events in patients who use cytotoxic agents of wide indication in different sites of oncological disease.

In this scenario of toxicity of antineoplastic drugs with important and disabling adverse dermatoneurological events, the role of nurses is to guide actions that promote self-care. The American Society of Clinical Oncology (ASCO) clinical practice guidelines state that there is no preventive agent to be recommended, however, some measures to promote safety can be adopted in the nursing consultation^(23)^.

Patient education about symptoms related to CIPN and HFS should be continuous. Establish a routine of nursing consultations, in order to assess the emergence/follow-up of symptoms, at each antineoplastic cycle, in addition to being beneficial to patients, it helps professionals to have a rigorous and scientific control of the signs and symptoms presented, in addition to the management of each one of them^(10)^. However, currently, validated instruments for CIPN assessment are, in most cases, not used in routine clinical monitoring ^(24)^. Because of this, a simple tool is needed, capable of early detecting symptoms to be used in clinical practice and in nursing consultations ^(25)^, especially for those with a lower level of education, which was observed that the chance of these patients spontaneously reporting symptoms of PN is lower ^(26)^.

Regarding the educational activities developed by nurses, defined in the method of this research, they are simple guidelines on daily care and patient routines that contribute to their perception of their treatment and management of adverse events. Through the nursing consultation, the professional has easy access to questions related to patients’ health-disease process, identifying possible predictive factors for the development of adverse dermatoneurological events. Thus, such information enhances the findings of Cormican and collaborator., 2018^(27)^, which emphasize nurses, within the multidisciplinary team, as responsible for maintaining the continuity of care, for knowing the processes related to the disease, the types of treatments and the possible adverse events^(28)^, in addition to being a link between the patient, the family and the multidisciplinary team, thus providing quality care^(29)^.

In the present study, the prevalence of males, the age group and the presence of some comorbidity, corroborate the research carried out at *Instituto Nacional de Câncer* (INCA - Brazilian National Cancer Institute), which describes that for males there is a higher frequency of PN, but in a chronic form. Regarding age, there is a prevalence of acute involvement in patients under 60 years of age and, among the comorbidities, diabetes is directly related to the development and worsening of PN. Moreover, some lifestyle habits, such as alcohol use, are also predictors of acute PN in patients using oxaliplatin^(30)^. In this study, although most participants are not alcoholics, the development of acute PN was not a predictor. However, it is suggested that health professionals observe and collect this information, since PN can be intensified when associated with alcohol consumption.

### Study limitations

The limitations of our study are related to therapeutic change due to disease progression and loss of follow-up. In this context, it is important to highlight that they are very frequent limitations in cancer studies. The use of a validated questionnaire for CIPN analysis that does not differentiate sensory and motor peripheral neuropathy can also be considered a limitation of this study.

### Contributions to nursing

The present study values nursing care for cancer patients by adopting specific tools that stimulate nursing actions and their participation in the results of anticancer treatment. In the clinical practice of oncology nursing, it is observed that the use of these tools are able to direct and identify the signs and symptoms of CIPN and HFS, thus contributing for these professionals to assist patients and evidence dermatoneurological adverse events during treatment with antineoplastic agents, providing guidelines and proposing nursing interventions for continuous improvement of care and thus contributing to the development of advanced practice in oncology nursing.

## CONCLUSIONS

In relation to dermatoneurological events, grade 1 HFS is not associated with interferences in ADL, while the presence of PN negatively affects ADL. With the cycles of treatment performed by patients, there is an increase in the intensity of neuropathic symptoms, which intensify their association with ADL. We emphasize the importance of identifying and monitoring PN and SMP in cancer patients undergoing treatment and that such events should be the target of oncology nurses’ performance, since they can influence the ADL of these patients and, consequently, the therapeutic response. Such results contribute to the clinical practice of oncology nursing, subsidize the development of advanced nursing practice and highlight the need for further studies on the implementation of tools and strategies that can minimize adverse events related to oncology therapy and that can enhance nursing interventions.
